# A novel *CLDN16* mutation in a large family with familial hypomagnesaemia with hypercalciuria and nephrocalcinosis

**DOI:** 10.1186/1756-0500-6-527

**Published:** 2013-12-10

**Authors:** Asma Deeb, Salima Atia Abood, Job Simon, Hormazdiar Dastoor, Simon HS Pearce, John A Sayer

**Affiliations:** 1 Endocrinology Department, Mafraq Hospital, AbuDhabi, United Arab Emirates; 2 Nephrology Department, Mafraq Hospital, AbuDhabi, United Arab Emirates; 3 Institute of Genetic Medicine, Newcastle University, International Centre for Life, Central Parkway, Newcastle upon Tyne, NE1 3BZ, UK

**Keywords:** CLDN16, Claudin-16, Hypercalciuria, Hypocalcaemia, Hypomagnesaemia, Nephrocalcinosis, End stage renal disease

## Abstract

**Background:**

Familial hypomagnesaemia with hypercalciuria and nephrocalcinosis is a rare tubulopathy leading to renal calcification and progressive renal failure.

**Case presentation:**

We report a consanguineous Arab family (of Qatari origin) with 7 affected siblings with variable phenotypes including hypomagnesaemia, hypercalciuria, nephrocalcinosis and renal stones. Presenting features included haematuria and recurrent urinary tract infections. As the biochemical and clinical phenotypes of this family resembled familial hypomagnesaemia with hypercalciuria and nephrocalcinosis, we performed genetic investigation in order to provide a precise molecular diagnosis. We screened all coding regions of the *CLDN16* gene and identified a novel mutation (c.G647A, p.R216H) which was found homozygously in the six severely affected cases, who manifested significant nephrocalcinosis, often nephrolithiasis and sometimes reduced GFR. Parents were both heterozygous for the mutation and, together with children carrying the mutation in its heterozygous state, exhibited mild or no biochemical phenotypes.

**Conclusion:**

Mutations in *CLDN16* underlie familial hypomagnesaemia with hypercalciuria and nephrocalcinosis but remain a rare cause of nephrocalcinosis and nephrolithiasis. Management includes reduction of hypercalciuria with thiazide diuretics, correction of serum magnesium and close monitoring of renal function given the significant risk of end stage renal failure with this inherited form of nephrocalcinosis.

## Background

Renal tubulopathies may present to a wide spectrum of clinicians with a variety of clinical, biochemical and radiological features. Renal tubular wasting of calcium and magnesium underlie the autosomal recessive disorder known as familial hypomagnesaemia with hypercalciuria and nephrocalcinosis (FHHNC) [1]. The clinical presentation often relates to the complications of severe hypercalciuria and nephrocalcinosis and may include haematuria, urinary tract infections and renal colic/calculi. The condition leads to progressive renal failure, often within the first two decades of life. Compound heterozygous or homozygous mutations in *CLDN16*, encoding a tight junction protein expressed in the thick ascending limb of the loop of Henle, underlie this disorder.

We report a large consanguineous family who presented with a variety of clinical and biochemical features suggestive of familial hypomagnesaemia with hypercalciuria and nephrocalcinosis (FHHNC) in whom we have identified a novel homozygous *CLDN16* mutation.

## Case presentation

**Figure 1:**
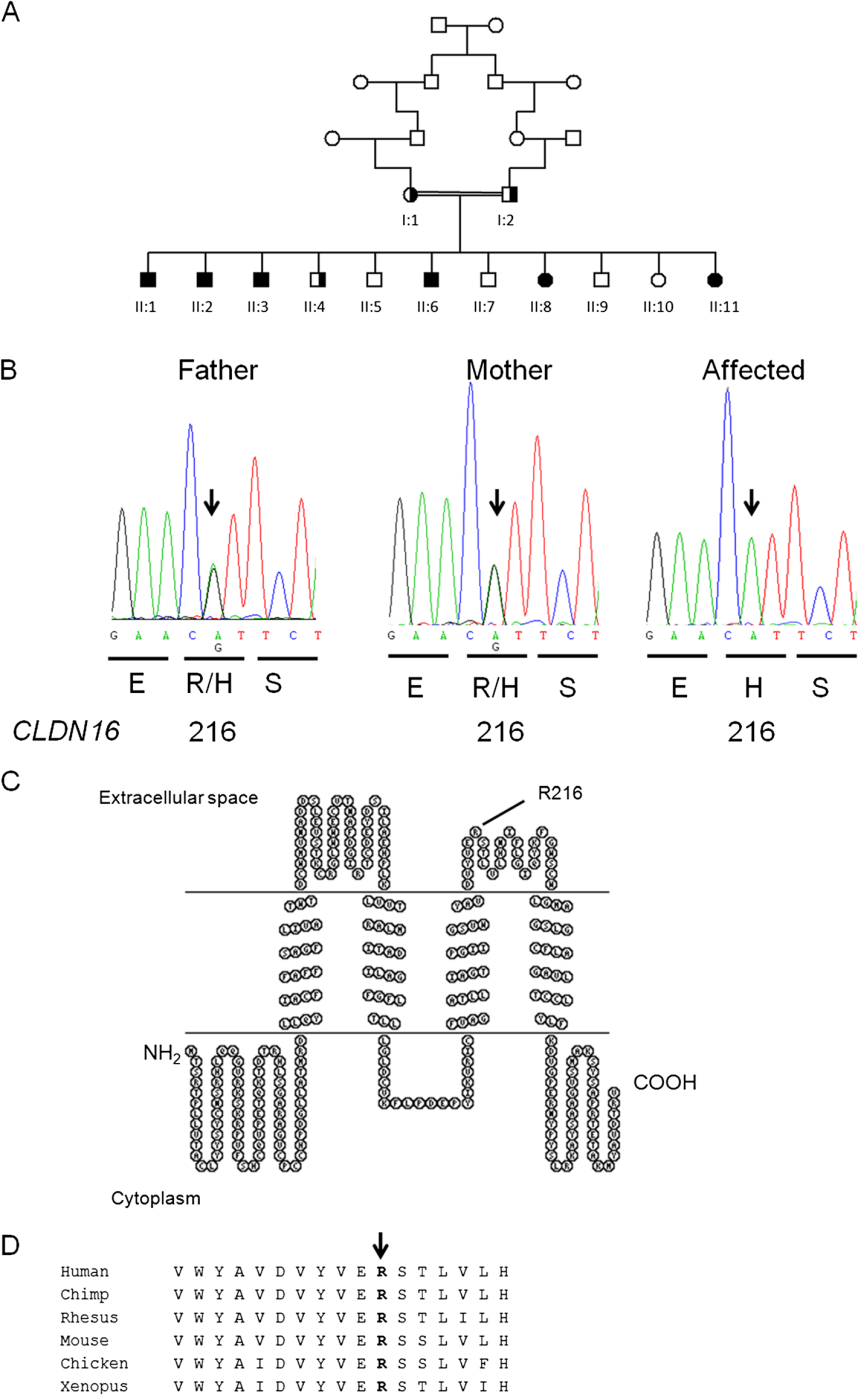
**Molecular genetic investigation of the family. (A)** Pedigree diagram of consanguineous multiplex family with homozygous and heterozygously affected members with Familial Hypomagnesaemia with Hypercalciuria and Nephrocalcinosis and *CLDN16* mutations. Circles females, squares males, shaded = affected, half shaded = heterozygous carrier. **(B)** Mutational analysis and segregation demonstrates a c. G647A nucleotide substitution in exon 4 of *CLDN16* leading to a missense change p. R216H. Amino acids and position are annotated below the chromatograms. **(C)** Claudin-16 protein (alias paracellin) model deduced from hydrophobicity plots and drawn using http://www.sacs.ucsf.edu/TOPO2. The mutated R216 in the second extracellular loop is annotated. **(D)** R216 residue (arrowed) is highly conserved from Human to *Xenopus*.

A large Qatari family (Figure 1A) presented with a variety of clinical features suggestive of an inherited tubulopathy. Clinical presentation of disease, even within the same family, was within a wide age range (Table 1).

**Table 1:** Clinical features at presentation

**Family member ID**	**Age**	**Sex**	**Presentation**	**Age of presentation**	**Serum calcium (Reference range 2.2-2.55 mmol/l)**	**Serum magnesium (Reference range 0.7-1.05 mmol/l)**	**25 (OH) vitamin D level (Reference range 75-250 mmol/l)**	**Hypercalciuria**	**Hypocitraturia**	**Nephrocalcinosis**	**Renal stone**	**GFR mls/min/1.73 m**^**2**^	**Genotype**
I:1	45	F		-	2.43	0.8	N/A	Yes	N/A	No	No	-	Het
I:2	48	M		-	2.40	N/A	N/A	No	N/A	No	No	-	Het
II:1	26	M	Macroscopic haematuria	20 years	2.11	0.52	35.1	Yes	Yes	Yes	Yes	48	Homo
II:2	25	M	Recurrent UTI, microscopic haematuria	20 years	2.10	0.60	N/A	Yes	Yes	Yes	No	37	Homo
II:3	24	M	Loin pain, passed a stone	17 years	2.13	0.59	29.4	Yes	N/A	Yes	Yes	67	Homo
II:4	23	M	Recurrent UTI, microscopic haematuria	17 years	2.25	0.85	21.3	No	N/A	No	No	126	Het
II:6	22	M	Recurrent UTI, microscopic haematuria	16 years	2.08	0.45	N/A	No	Yes	Yes	No	37	Homo
II:8	12	F	Loin pain	7 years	2.09	0.47	35.2	No	Yes	Yes	No	36	Homo
II:11	3	F	Haematuria	17 months	2.40	0.76	61.0	No	N/A	Yes	No	N/A	Homo

F, female; GFR, glomerular filtration rate; Het, heterozygous mutation; Homo, homozygous mutation; M, male; N/A, not available; UTI, urinary tract infection.

The index case (II:1) presented 20 years of age with macroscopic haematuria. He had a past medical history of recurrent attacks of left loin pain typical of renal colic and was known to have renal stone disease and recurrent urinary tract infections. Clinical examination revealed normal blood pressure and a BMI of 28.5 Kg/m^2^.

Biochemical investigations revealed hypercalciuria (24 h urine calcium 9.83 mmol/day; reference range 2.5-8.5), hypomagnesaemia (0.52 mmol/l) and hypocalcaemia (2.11 mmol/l). Serum parathyroid hormone was raised at 16.96 pmol/L (reference range 1.3-6) in the context of low total vitamin D levels (39.9 nmol/l; reference range 75-200). There was also profound hypocitraturia (21 mg/day, reference range 290-888). Serum creatinine was elevated at 160 umol/l consistent with CKD stage 3 (Table 1).

**Figure 2:**
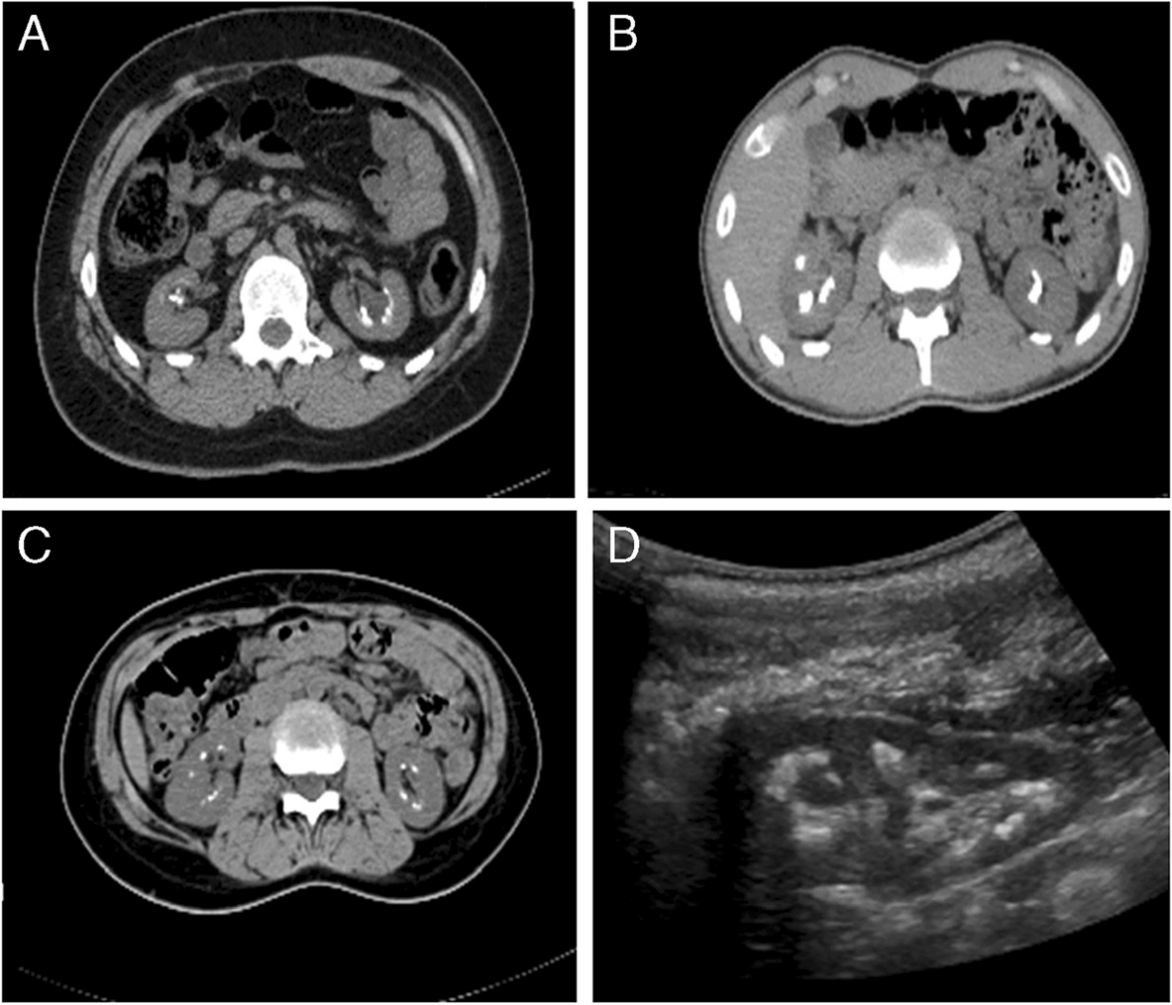
**Imaging of the renal tract in affected members. A**. CT scan showing bilateral nephrocalcinosis in II:1, aged 26 years. **B**. CT scan showing bilateral nephrocalcinosis in II:3, aged 22 years. **C**. CT scan showing bilateral nephrocalcinosis in II:8, aged 12 years. **D**. Renal USS demonstrating nephrocalcinosis in II:11, aged 3 years.

A renal ultrasound demonstrated multiple fine echogenic cortical calcifications in both kidneys consistent with nephrocalcinosis and parenchymal renal calculi. The right kidney measured 9.8 cm in length and the left kidney measured 10.0 cm. No cystic change was noted within the kidneys. An abdominal CT confirmed nephrocalcinosis (Figure 2A). Isotope imaging of the neck showed no evidence of a parathyroid adenoma.

The patient was managed with an increase in fluids, to reduce risk of stone formation and the addition of magnesium supplements and a thiazide diuretic in the form of hydrochlorothiazide 25 mg daily.

A second family member (II:8) presented at 7 years of age with right sided loin pain. Biochemical investigations also revealed hypocalcaemia (2.09 mmol/l) and hypomagnesaemia (0.56 mmol/l) with a raised PTH 11 pmol/l in the context of a low total vitamin D level (36 nmol/l). There was no evidence of hypercalciuria or hypermagnesuria (Table 1). Renal imaging (USS and CT scan) demonstrated nephrocalcinosis with bilateral symmetrical diffuse calyceal calcifications (Figure 2C). The child was similarly treated with magnesium supplementation and thiazide diuretics.

The discovery of these two cases of hypocalcaemia and hypomagnesaemia with hypercalciuria and nephrocalcinosis within the same family prompted a review of other affected family members, who had presented independently to other physicians and their parents. The clinical findings are detailed in Table 1 and renal imaging is shown in Figure 2. Family member II:11 is noteworthy given the early presentation at 17 months of age, following a urinary tract infection. USS of the renal tract, even at this young age, demonstrated nephrocalcinosis (Figure 2D).

### Genetic investigations

Following informed consent from all adult patients/relatives and from the parents or guardians of the patients who were under the age of 18, DNA samples were obtained and all coding regions of the *CLDN16* gene were amplified and sequenced directly using Sanger sequencing.

We identified a novel missense mutation in exon 4 of the *CLDN16* gene (c.G647A, p.R216H), located in the second extracellular loop (Figure 1B, C). *In silico* prediction tools predict that the mutation is pathogenic. The variant was absent from both the 1000 Genomes and Exome Variant Server databases. The Arginine residue is highly conserved (Figure 1D). Interestingly, a R216C mutation, affecting the same residue, has been previously reported [2,3]. This R216C sequence variant was not observed in 100 European and 50 Japanese control alleles [2,3].

Mutations in *CLDN16* (alias *PCLN1*) cause the autosomal recessive condition familial hypomagnesaemia with hypercalciuria and nephrocalcinosis (FHHNC) [1]. The phenotype includes renal magnesium and calcium wasting secondary to defects in the thick ascending limb reabsorption of calcium and magnesium, which leak through defective tight junctions owing to mutations in *CLDN16* encoding claudin-16. The expression of claudin-16 is limited to the tight junctions of renal epithelial cells of the thick ascending limb of the loop of Henle [4]. The clinical presentation may be in childhood or adolescence with symptomatic hypocalcaemia [3,5-8].

As seen in this family, nephrocalcinosis and recurrent nephrolithiasis are also seen. Nephrocalcinosis may contribute to a renal tubular acidification defect and to the inability to concentrate urine and hypokalaemia may also be a feature. Our family was typical of many cases of FHHNC, with affected members presenting with recurrent urinary tract infections. Other features can include polyuria/polydipsia, rickets, haematuria, muscular tetany, seizures, failure to thrive, vomiting, and abdominal pain [9,10]. Additional biochemical findings can include hypocitraturia [11], increased parathyroid hormone (PTH) levels (independent of GFR) [5], and hyperuricaemia. Worryingly, FHHNC is often complicated by progressive renal failure which occurs during childhood or adolescence. Within this family there was a variable loss of renal function, with some family members with significant renal impairment at a young age (Table 1).

The phenotype of heterozygous carriers of *CLDN16* mutations is not totally benign. Hypercalciuria, as seen in this family, and even nephrolithiasis may be seen in heterozygous “carriers” of mutations [5,10].

Mutations in the *CLDN19* gene (encoding a related tight junction protein, Claudin-19) can produce the same biochemical phenotype as *CLDN16* mutations [3]. However, patients with *CLDN19* mutations had additional phenotypes including eye involvement and severe visual impairment.

Mutational analysis has allowed the identification of ~50 different mutations of *CLDN16*. In one study of 32 patients, nearly half had a L151F missense mutation. This mutational hot spot was proposed to be due to a founder effect (Germany and Eastern European countries) [10]. The R216H mutation, affecting the second extracellular loop, found in this family is novel. The extracellular loops are important for interactions between claudin molecules of neighbouring cells [12]. Mutations affecting the same amino acid have been described previously. An R216C mutation has been described in a heterozygous state, in combination with a R149Q missense mutation in a Japanese child presenting at 5 years of age with haematuria, hypercalciuria, and nephrocalcinosis [2,3]. In addition, a R216T mutation has also been described [4,13]. In functional studies the R216T mutation was shown to mis-traffic to the tight-junction [13] although was seen to correctly localise to the tight junction, with a magnesium transport defect, by other investigators [4].

Other inherited disorders that may present in childhood with hypomagnesaemia include Gitelman’s syndrome [14], familial hypomagnesaemia with secondary hypocalcaemia (HSH) [15] and autosomal dominant hypomagnesaemia [16]. Gitelman’s syndrome is renal salt-wasting alkalosis characterised by a hypokalaemic metabolic alkalosis [14]. Gitelman’s patients typically have hypocalciuria and do not exhibit nephrocalcinosis [17]. Mutations in *SLC12A3* encoding the thiazide-sensitive sodium-chloride co-transporter underlie this disease [14]. Familial hypomagnesaemia with secondary hypocalcaemia (HSH) is a rare autosomal recessive disease caused by mutations in *TRPM6* an apical magnesium uptake channel in the intestine and distal convoluted tubule of the kidney [18]. The biochemical phenotype usually reveals an extremely low serum magnesium. There is secondary low serum calcium levels, thought to be due to an impaired synthesis and/or release of PTH. Again, nephrocalcinosis would not be an expected feature of patients with *TRPM6* mutations. Autosomal dominant hypomagnesaemia is a renal magnesium wasting tubulopathy. A mild hypomagnesaemia occurs and an associated hypocalciuria. Mutations in the *FXYD2* gene are responsible for this disorder [16].

Another condition that may cause diagnostic confusion with FHHNC is autosomal dominant hypocalcaemia (ADH) secondary to activating mutations of the calcium-sensing receptor that lead to hypocalcaemia with hypercalciuria [19]. Hypomagnesaemia can be a feature of this condition and typically serum PTH is in the low-normal range. Here the inheritance pattern is autosomal dominant and the majority of patients are asymptomatic and are diagnosed as adults when hypocalcaemia is noted as an incidental finding. Some patients exhibit a salt wasting phenotype with hypokalaemia, mimicking Bartter’s syndrome [20]. In ADH, vitamin D and calcium supplementation worsen the hypercalciuria, and may lead to increased nephrocalcinosis and calcium stone formation. Genetic testing of the *CASR* gene allows a molecular diagnosis to be made [21].

For the medical management of cases of FHHNC, attention should be given to the management of the biochemical abnormalities. Thiazide diuretics can reduce effectively the calcium excretion in patients with FHHNC [22] and magnesium supplementation should be given. In addition, Vitamin D and calcium supplementation may be required. There was evidence of vitamin D deficiency in the family we describe. Limited sunlight exposure and poor oral intake of vitamin D are common risk factors for vitamin D deficiency in the Middle East [23]. The dramatic loss of renal function in patients with *CLDN16* mutations is a noteworthy and worrying feature, and the aetiology of which is currently unclear [3]. It can be speculated that the dramatic nephrocalcinosis is contributory, yet there are other renal disorders with nephrocalcinosis that do not show similar rates of decline in renal function [24]. Strategies to measure and optimise renal function are important. Urinary tract infections and obstructive calculi should be treated promptly to prevent ascending infections, obstruction and further renal insults. As expected from an inherited tubulopathy, following renal transplantation, magnesium and calcium regulation normalize, indicating that FHHNC patients are ideal candidates for renal transplantation [5].

## Conclusions

In summary, in we have made a molecular genetic diagnosis of a *CLDN16* mutation in a large consanguineous family with nephrocalcinosis, renal stones and renal impairment. Age and clinical features at presentation can be very variable, even from within the same family. A molecular genetic diagnosis enables a screen of other family members at risk of disease and allows management of urinary electrolyte abnormalities to prevent further deterioration in renal function and renal stone formation.

## Consent

Written informed consent to publish this case report was obtained for all adult patients, and from the parents or guardians of the patients who were under the age of 18. A copy of the written consent is available for review by the Editor-in-Chief of this journal.

## Competing interests

The authors declare that they have no competing interests.

## Authors’ contributions

AD carried out clinical data reviews and drafted the manuscript; SAA, JS, and HD participated in the coordination of the clinical and radiological data and helped draft the manuscript; SHSP and JAS conceived the study, performed sequence analysis and drafted the manuscript. All authors read and approved the final manuscript.
